# Effect of lactic acid bacteria, yeast, and their mixture on the chemical composition, fermentation quality, and bacterial community of cellulase-treated *Pennisetum sinese* silage

**DOI:** 10.3389/fmicb.2022.1047072

**Published:** 2022-10-27

**Authors:** Chaosheng Liao, Xiaolong Tang, Maoya Li, Guangrou Lu, Xiaokang Huang, Lin Li, Mingjie Zhang, Yixiao Xie, Chao Chen, Ping Li

**Affiliations:** ^1^College of Animal Science, Guizhou University, Guiyang, China; ^2^Key Laboratory of Animal Genetics, Breeding and Reproduction in the Plateau Mountainous Region, Ministry of Education, Guizhou University, Guiyang, Guizhou, China

**Keywords:** biological pretreatment, lactic acid bacteria, yeast, bacterial community, cellulose, *Pennisetum sinese*

## Abstract

The present study investigated the effects of *Lentilactobacillus buchneri*, *Saccharomyces cerevisiae*, and a mixture of the two on the cellulose degradation and microbial community of cellulase-treated *Pennisetum sinese* (CTPS) during biological pretreatment. The CTPS was stored without additives (CK) or with *L. buchneri* (L), yeast (Y, *S. cerevisiae*), and their mixture (LY) under anaerobic conditions for 60 days. All inoculants enhanced the anaerobic fermentation of CTPS. In relative to L, inoculations with Y and LY decreased the cellulose level of fermented-CTPS by 8.90 ~ 17.13%. Inoculation with L inhibited the growth of *Weissella cibaria* during anaerobic storage. However, inoculations with LY increased the relative abundance of the homofermentative bacterium *Lactiplantibacillus plantarum* by 6.04%. Therefore, inoculating *S. cerevisiae* reduced the adverse effects of *L. buchneri*-stimulated fermentation on cellulose degradation by altering the bacterial community during anaerobic storage of *P. sinese.* This work provides a new insight for the subsequent anaerobic digestion of *P. sinese*.

## Introduction

*Pennisetum sinese*, widely known as King grass, a widely used forage crop in the world, is a fast-growing gramineous grass with large biomass and has recently gained increasing attention as an energy crop (Li et al., 2018a). Anaerobic digestion (AD) is known as a widely used and cost-effective way to convert agriculture biomass into biogas (Wu et al., 2019). However, the majority of *P. sinese* are used for forage utilization and only a few are used for biomass energy production, which is because the lignocellulose in *P. sinese* is difficult to be decomposed during AD (Patinvoh et al., 2017). Therefore, pretreatment of *P. sinese* to improve the efficiency of AD is an essential step.

Until now, biological pretreatment of lignocellulose has been extensively investigated, such as enzymatic, fungal, composting and ensiling pretreatment (Wang et al., 2020). Among them, the ensiling pretreatment process is again accompanied by the addition of microorganisms, enzymes. Cellulase enzyme is a biological additive often used to pretreat high-fiber plant materials due to their ability to degrade structural carbohydrates to soluble sugars, which act as substrates for subsequent lactic acid bacteria anaerobic fermentation (Colombatto et al., 2004). However, multiple factors, such as enzyme type, concentration, and activity, application method, target substrate, and attached microorganisms of raw materials, govern cellulase activity (Li et al., 2018b). Moreover, the application of cellulase to *P. sinese* does not always present desirable efficiency due to the enzyme instability during pretreatment (Moharrery et al., 2009). Therefore, the degradation of cellulose by cellulases under microbial conditions is controversial. However, the role of microorganisms in enzyme-driven fiber degradation has rarely been reported. Exploring the effect of cellulase degradation of cellulose under microbial conditions will help to improve the efficiency of subsequent AD of *P. sinese.*

Yeast and lactic acid bacteria (LAB) inoculants have been used as additives during the ensilage of fresh materials, as they preserve the nutritional qualities of the material effectively. Matano et al. (2013) found that yeast mitigated the irreversible adsorption of cellulase onto crystalline cellulose and increased cellulase activity. It means that the presence of yeast may increase the degradation of cellulose during biological pretreatment. However, the role of yeast in promoting cellulose degradation during biological pretreatment has been rarely explored. Meanwhile, *Lentilactobacillus buchneri* is a heterotrophicus LAB often used as a bacterial inoculant in ensiling pretreatment due to its ability to produce volatile fatty acids, such as acetate and propionate from lactate (Kung et al., 2018). This conversion increases biogas emissions during AD. Stokes (1992) found that the interaction of LAB with cellulase is antagonistic. This implies that inoculation withe LAB maybe inhibits the degradation of cellulose. However, LAB inoculants are almost invariably applied with enzyme additives, making it difficult to differentiate between bacterial and enzyme-mediated ensiling responses (Xu et al., 2017). Few studies have been conducted to investigate the changes in the fiber fraction during biological pretreatment with a combination of cellulose, yeast, and LAB. Moreover, the fiber composition not just has an impact on the efficiency of AD, but also on the subsequent methane production, as cellulose can be degraded and converted to methane (Fujiwara et al., 2022). Therefore, it is essential to investigate the combined effect of microorganisms and cellulases to improve the AD efficiency of *P. sinese*.

The present study investigated the effects of *L. buchneri*, *Saccharomyces cerevisiae* and their mixtures as pretreatment inoculations on the fiber fraction and bacterial community of cellulase-treated *P. sinese* (CTPS). We hypothesized that the inoculation of *L. buchneri* might affect cellulase activity and inhibit cell wall degradation in *P. sinese* while adding *S. cerevisiae* would undo this effect.

## Materials and methods

### Sample preparation

For this study, *P. sinese* was harvested from the yellow cow breeding base in Fenggang County (27°42′ N, 106°55′ E), Zunyi City, Guizhou Province, China. The *P. sinese* plants were chopped cm to 1–3 cm in length and randomly divided into four blocks with 25 replicates per treatment. The forage from each block was first pretreated with cellulase (F; 2 × 10^2^ μ/g FM, Shanghai Macklin Biochemical Co., Ltd., Shanghai, China, activity, 50 U/mg) and subsequently treated as follows: (i) no additive (CK); (ii) *L. buchneri* (L; 10^5^ cfu/g FM, Xi’an Jushengyuan Biotechnology Co., Shaanxi, China); (iii) yeast (Y; *S. cerevisiae*, 2 × 10^5^ cfu/g FM, Xi’an Jushengyuan Biotechnology Co., Shaanxi, China); and their mixture (LY). The inoculations were diluted with sterile water and sprayed evenly on the cellulase-treated *P. sinese* (CTPS), and CK treated with equal quantities of sterile water. Then, 100 g of CTPS uniformly mixed with each inoculant was manually loaded into a 500 ml Storage Jar (4 treatments ×25 Storage Jar), connected to a collection bag and sealed the jars after tightly vacuumed with an evacuator (SHZ-III type water Circulating Vacuum Pump, Yarong Biochemical Instrument Factory, Shanghai, China). Five Storage Jar per treatment were opened and sampled after 7, 14, 30, 45, and 60 days of storage. A total of 100 samples (4 treatments × 5 storage periods × 5 replicates) were collected and analyzed for fermentation quality, chemical composition and bacterial community composition during fermentation.

### Chemical composition analysis

Each storage sample of 10 g was uniformly mixed with 90 ml of sterile water in a laboratory juicer for 1 min and filtered through four layers of gauze. The filtrate was then centrifuged at 4,500 × *g* for 15 min at 4°C. High-performance liquid chromatography (HPLC) was used to evaluate the concentration of butyric, propionic, acetic, and lactic acids (Li et al., 2019). The method of Broderick and Kang (1980) was used to determine the concentration of ammoniacal nitrogen. A pH meter was used to determine the pH of the sample solution.

Each storage sample (70 g) was dried at 65°C for a constant weight to determine the dry matter (DM) content, and then ground through 0.20 mm-mesh sieves for analysis of chemical components. Crude protein (CP) content was determined following the AOAC (1990) method. Neutral detergent fiber (NDF) and acid detergent fiber (ADF) content were determined according to the method described by Van Soest et al. (1991). The method of McDonald et al. (1991) was used to ascertain the content of water-soluble carbohydrates (WSC).

### Bacterial community analysis

To determine the identities of the species present, we used the CTAB method to extract the total genomic DNA from each storage sample. After purification, DNA samples were diluted to 1 ng ml^−1^ with sterilized water. We amplified the full-length 16S ribosomal RNA (rRNA) gene using specific, barcoded primers (1514R and 27F; Yan et al., 2019). Polymerase Chain Reaction (PCR) amplification was conducted using TransStart®FastPfu DNA Polymerase (TransGen Biotech Co., Ltd., Beijing, China) and the PCR products purified using the QIAquick Gel Extraction Kit (QIAGEN LLC., Germantown, MD, United States). DNA libraries were generated with the SMRTbell Template Prep Kit (PacBio, Menlo Park, CA, United States) and sequenced using the PacBio Sequel system. In order to annotate taxonomic information and assess phylogenetic relationships with the Silva SSUrRNA Database, we used Novogene Bio-Technology Co., Ltd. (Beijing, China) to process raw sequences. Functional prediction, principal coordinates analysis (PCoA), and alpha diversity of the microbial community were assessed using the NovoMagic platform (Novogene Bio-Technology Co., Ltd., Beijing, China).

### Statistical analysis

Data of changes in chemical composition, microbial population and bacterial community indices during storage was repeatedly compared with Duncan’s test, using the SPSS program version 26.0 (IBM Corp., Armonk, NY, United States). Differences were considered statistically significant only when the probability level was lower than 0.05 (*p* < 0.05). In addition, Spearman correlation was analyzed among bacterial community compositions and anaerobic fermentation parameters.

## Results and discussion

### Chemical composition of CTPS before and after anaerobic storage

Table 1 shows the chemical composition of fresh *P. sines*e before fermentation. The DM of *P. sinese* was 20.74%, and the NDF and ADF content were 61.34% DM and 35.22% DM, respectively. The WSC content was 5.65% DM, which is close to the WSC content (5.73% DM) reported by Li et al. (2018a). Typically, the WSC of the fresh material acts as an important substrate for silage fermentation, and a material with a WSC content >5% DM contributes to good silage quality (Cai et al., 1998). Thus, the WSC content of fresh *P. sinese* was sufficient for silage fermentation.

**Table 1:** Chemical composition of cellulase-treated *Pennisetum sinese* (CTPS) *Pennisetum sinese* before anaerobic fermentation.

Items	*Pennisetum sinese*
pH	5.49 ± 0.03
DM, %FM	20.74 ± 0.43
WSC, %DM	5.65 ± 0.08
CP, %DM	7.42 ± 0.16
NDF, %DM	61.34 ± 1.13
ADF, %DM	35.22 ± 1.08

ADF, acid detergent fiber; CP, crude protein; DM, dry matter; FM; fresh matter; NDF, neutral detergent fiber.

Table 2 shows the chemical composition of CTPS after 60 days of fermentation. The DM content did not show differed significantly between the samples. The degradation of CP was mainly initiated by microbial and plant enzymes. In this experiment, the various CTPS samples after fermentation with different inoculants showed no significant difference in the CP content. This is due to the failure of high pH (>4.2) to inhibit microbial degradation of CP. Meanwhile, L and Y inoculated CTPS had higher NDF and ADF contents than CK (CTPS was not inoculated with microbial fermentation) after 60 days of fermentation, this may be attributed to the consumption of available nutrients stimulating an increase in NDF and ADF during anaerobic fermentation (Li et al., 2022). However, the LY-treated CTPS had the lowest NDF (53.49% DM) and ADF (29.70% DM) content, probably due to the LY inoculum promoting cell wall degradation by cellulase. Exogenous cellulolytic enzymes are usually added to the woody fiber material before ensiling. These cellulases degrade cellulose and produce WSC to facilitate anaerobic fermentation. In CTPS, the L treatment (30.77% DM) and Y treatment (28.03% DM) samples had higher cellulose content than CK treated samples (26.33% DM). We attribute this phenomenon to the different effects of *L. buchneri* and *S. cerevisiae* on cellulase. Interestingly, the LY-treated (25.50% DM) samples showed a different result from it. Earlier, Chen et al. (2020) reported that a mixture of *Lactiplantibacillus plantarum*, *L. buchneri* and cellulase promoted the degradation of fiber fractions. However, Stokes (1992) found that inoculation with a multispecies homofermentative LAB culture (*L. plantarum*, *Levilactobacillus brevis*, *Pediococcus acidilactici*, *Streptococcus cremoris*, and *Streptococcus diacetylactis*) was antagonistic to cellulase. This indicated that different microorganisms have different effects on cellulase. Therefore, we believe that the phenomenon observed in this study may be due to the inoculated *S. cerevisiae* and *L. buchneri* affecting the cellulase activity, which further affects the degradation of the fiber fraction. The higher ADL content in the samples treated with LY supports this fact.

**Table 2:** Chemical composition of cellulase-treated *Pennisetum sinese* (CTPS) after 60 days of anaerobic fermentation.

Items	Treatments				SEM	*p*-value
	CK	L	Y	LY		
DM, %FM	18.85	19.23	18.44	18.03	0.33	0.668
CP, %DM	8.23	8.75	8.65	8.60	0.12	0.46
NDF, %DM	55.19^b^	59.85^a^	58.36^a^	53.49^b^	0.84	0.002
ADF, %DM	29.72^b^	34.69^a^	31.76^b^	29.70^b^	0.71	0.009
Hemicellulose, %DM	25.46	25.17	26.59	23.79	0.45	0.179
ADL, %DM	3.39^c^	3.91^ab^	3.72^bc^	4.18^a^	0.07	0.011
Cellulose, %DM	26.33^b^	30.77^a^	28.03^b^	25.50^b^	0.69	0.005

The CTPS was treated without (CK) or with *Lentilactobacillus buchneri* (L), yeast (Y), and a mixture of *L. buchneri* and yeast (LY). SEM, standard error of the mean; DM, dry matter; FM; fresh matter; CP, crude protein; NDF, neutral detergent fiber; ADF, acid detergent fiber; ADL, acid detergent lignin.

^a–c^Means within the same row with different letters are significantly different (*p* < 0.05).

### Fermentation profile of CTPS during anaerobic storage

We further analyzed the fermentation characteristics of CTPS during storage (Table 3). The interactive effect of pH was not significant during the anaerobic fermentation. However, the pH of the L-treated CTPS was the lowest (4.34) after 60 days of fermentation, which was strongly related to the production of lactic acid by the inoculant *L. buchneri*. Similarly, Zielinska et al. (2015) reported that *L. buchneri* inoculation decreases the pH of alfalfa silage.

**Table 3:** Fermentation profile of cellulase-treated *Pennisetum sinese* (CTPS) during anaerobic storage.

Items	Treatments (T)	Storage period (D)					SEM	*p*-value		
		Day 7	Day 14	Day 30	Day 45	Day 60		*T*	*D*	*T × D*
pH	CK	4.52	4.47	4.73	4.57	4.56	0.02	0.004	<0.001	0.092
	L	4.45	4.38	4.52	4.58	4.34				
	Y	4.45	4.20	4.56	4.58	4.45				
	LY	4.46	4.38	4.92	4.74	4.41				
Ammonia-N % TN	CK	1.37Be	2.19 Bd	4.21Ac	5.76ABb	9.63Aa	0.09	0.276	<0.001	0.002
	L	1.81ABc	2.53BAc	5.31Ab	6.81Aab	8.01ABa				
	Y	2.22Ac	3.15Ac	4.55Ab	4.58Bb	7.41Ba				
	LY	2.23Ac	2.95BAc	4.70Ab	5.14ABb	8.20ABa				
WSC % DM	CK	3.73Ba	3.29Bb	2.86ABc	2.56Ad	1.88e	0.06	0.012	<0.001	0.005
	L	5.33Aa	4.86Aa	2.71Bb	2.14ABb	1.96b				
	Y	5.11Aa	3.93ABb	3.45Ab	2.09ABc	1.93c				
	LY	4.73ABa	4.01ABb	3.04ABc	1.92 Bd	1.70d				
Lactic acid % DM	CK	1.33 Bd	2.05Cc	2.67Bb	2.45b	3.03Ba	0.03	<0.001	<0.001	<0.001
	L	0.70Cd	2.92Bb	3.14Ab	2.16c	5.04Aa				
	Y	0.86Cc	3.29Aa	3.29Aa	2.46b	3.30Ba				
	LY	1.77Ad	3.15ABab	2.61Bbc	2.34 dc	3.62Ba				
Acetic acid % DM	CK	0.51c	0.47Bc	2.89Aab	2.44Ab	3.23ABa	0.04	<0.001	<0.001	<0.001
	L	0.32d	0.91Ac	1.19Cbc	1.60BCb	3.48Aa				
	Y	0.42c	0.90Ab	1.96Ba	2.37ABa	2.39Ba				
	LY	0.34d	0.64 Bd	2.23Bb	1.32Cc	3.38Aa				
Propionic acid % DM	CK	ND	0.07	0.39	0.25	0.41	0.02	0.143	<0.001	0.291
	L	ND	0.07	0.24	0.17	0.53				
	Y	ND	0.16	0.26	0.25	0.19				
	LY	ND	0.11	0.41	0.48	0.48				
Butyric acid % DM	CK	ND	ND	ND	ND	0.07	0.03	0.447	0.293	0.549
	L	ND	ND	ND	ND	ND				
	Y	ND	ND	ND	ND	ND				
	LY	ND	ND	ND	ND	0.51				

The CTPS was treated without (CK) or with *Lentilactobacillus buchneri* (L), yeast (Y), and a mixture of *L. buchneri* and yeast (LY); SEM, standard error of mean; WSC, water-soluble carbohydrates; ND, not detected; ^A-C^ indicate differences between values in the same column at *p* < 0.05; ^a-g^ indicate differences between values in the same row at *p* < 0.05.

The WSC content is a limiting factor for fermentation. Generally, a minimum WSC content of about 3% DM is necessary to successfully preserve material (Zhang et al., 2010). However, WSC, the primary substrate for microbial growth, decreases gradually with fermentation progress. CK treated CTPS had the fastest decrease in WSC content at 7 days of anaerobic storage (5.65% DM to 3.73% DM), and the predominant inoculants inhibited the consumption of WSC by undesirable bacteria during the anaerobic storage. Guo et al. (2014) found more residual WSC in *Lactobacillus-*treated and fibrolytic enzyme-treated forages, which was *Lactobacillus* inhibits the consumption of WSC by undesirable bacteria. Besides, the inoculants delayed the decrease in WSC content of anaerobic fermentation compared with CK. Similarly, Li et al. (2022) also reported that the LAB inoculation delayed the decline in WSC. A high level of ammonia-N (>10% of total N) in sample indicates excessive protein breakdown, usually caused by a slight decrease in pH and/or *Clostridium* fermentation (Kung et al., 2018). In the present study, ammonia-N increased with the fermentation progress. The lowest ammonia-N level was detected in the Y-treated CTPS compared with the control and other additives, indicating good preservation with *S. cerevisiae* as the inoculant. This may be the result of the inoculated *S. cerevisiae* inhibiting the growth of NH3-N producing bacteria, thus reducing the NH3-N content in the silage. However, the mechanism underlying the slow degradation of protein to ammonia in the presence of *S. cerevisiae* is unclear.

Lactic acid is considered responsible for the decrease in pH. In this study, lactic acid increased in all CTPS samples, followed by a decrease after 45 days of anaerobic storage, with a maximum at the subsequent time. The changes in lactic acid concentration explain the fluctuation in pH. Notably, at 7 days, the LY-treated CTPS had the highest lactic acid content (1.77% DM). Earlier, Li et al. (2017) also reported that the combination of LAB and cellulase increased the concentration of lactic acid, while Mu et al. (2020) explained that cellulase indirectly provided LAB fermentable sugars by degrading cellulose, subsequently increasing the lactic acid level. Additionally, Carvalho et al. (2021) found that a few yeasts had the potential to produce amylase, cellulase, and protease. Therefore, we attributed the higher lactic acid level in the LY-treated CTPS to the cellulases. We also believe these cellulases led to the lowest cellulose content in the LY-treated CTPS at 60 days. However, the lactic acid level of the LY-treated CTPS lowered than the L-treated CTPS at 60 days, which may be due to the continuous consumption of lactic acid by inoculated yeast (Dolci et al., 2011). Meanwhile, the acetic acid content gradually increased with increasing fermentation time, consistent with Zhou et al. (2016). Studies have shown that the inoculation of heterologous *L. buchneri* increases fermentation’s acetic acid level (Huang et al., 2021). However, in the L-treated CTPS after 60 days of anaerobic storage, the acetic acid content was not significantly different from the CK-treated CTPS (*p* > 0.05), probably due to the higher lactic acid that inhibited acetic acid production (Lin et al., 2021). Meanwhile, the low acetic acid levels in the Y-treated CTPS at 60 days could be attributed to the continuous consumption of acetic acid in other bacteria (Ogunade et al., 2017), which will subsequently burden bioenergy production. Studies have proven the presence of propionic acid bacteria that convert glucose and lactic acid to propionic acid and acetic acid (Li et al., 2022). Therefore, the propionic acid produced after 7 days of fermentation in CTPS may be due to the action of these propionic acid bacteria. Under unfavorable conditions, certain undesirable microorganisms, such as *Clostridium*, convert lactic acid to butyric acid (Steinbrenner et al., 2019). The butyric acid in the LY and CK-treated CTPS may be due to the fermentation of *Clostridium*. These results suggest that the LY-treated CTPS increased lactic acid content by enhanced cellulose degradation in pre-fermentation, however, the mechanisms behind this need to be explored.

### Bacterial diversity of CTPS during anaerobic storage

Furthermore, we analyzed the alpha diversity of bacteria found in CTPS after anaerobic fermentation (Table 4). The coverage value of all samples was above 99%, indicating that sequencing adequately captured most of the bacterial communities. The observed species number increased in CTPS during the 60 days anaerobic fermentation. Similarly, Yan et al. (2019) reported an increase in species after anaerobic fermentation of ryegrass. Shannon’s and Simpson’s diversity indices varied similarly among the treatments. The alpha diversity at 60 days was the lowest in the LY-treated CTPS, probably due to the combined effect of *S. cerevisiae* and *L. buchneri.* Usually, a low bacterial community diversity is due to the increased abundance of dominant bacteria (Wayne Polley et al., 2007). Meanwhile, ACE and Chao1 are used to evaluate the richness of the microbial community. The richness indices increased in all samples after 60 days of storage, consistent with Ren et al. (2019), who reported an increase in the bacterial richness during anaerobic fermentation of top sugarcane.

**Table 4:** Bacterial alpha diversity of cellulase-treated *Pennisetum sinese* (CTPS) during anaerobic storage.

Items	Fresh forage	Treatments (T)	Storage period (D)					SEM	*p*-value		
			Day 7	Day 14	Day 30	Day 45	Day 60		*T*	*D*	*T × D*
Observed species	147	CK	231.33	217.00	170.67	213.00	218.00	6.1	0.935	0.005	0.249
		L	216.67	225.33	145.33	207.33	221.33				
		Y	138.33	270.67	146.67	196.33	242.33				
		LY	161.33	230.33	214.33	173.67	239.33				
Shannon	3.53	CK	4.13Aa	3.47b	2.96b	3.34Ab	3.58ab	0.05	0.275	<0.001	0.02
		L	3.78ABa	3.56ab	2.68c	3.09ABbc	3.43ab				
		Y	3.29Cab	4.36a	2.77b	2.62Bb	3.62ab				
		LY	3.35BCa	3.41a	3.20a	2.71Bb	3.43a				
Simpson	0.82	CK	0.85Aa	0.73bc	0.63c	0.73Abc	0.77ab	0.01	0.254	<0.001	0.02
		L	0.81ABa	0.76ab	0.62c	0.66ABbc	0.75ab				
		Y	0.77Bab	0.84a	0.66bc	0.57Bc	0.76ab				
		LY	0.77Ba	0.72ab	0.69b	0.60Bc	0.73ab				
Chao1	151.86	CK	254.54	243.85	185.75	238.97	241.80	7.16	0.965	0.01	0.296
		L	242.96	251.78	165.24	231.27	246.43				
		Y	154.50	303.00	164.88	225.71	267.80				
		LY	179.66	266.34	249.51	196.25	263.33				
ACE	155.29	CK	258.67	244.93	188.34	240.63	242.34	7.10	0.981	0.007	0.236
		L	243.33	255.89	169.59	234.89	251.44				
		Y	153.09	308.90	168.88	231.61	275.68				
		LY	181.14	271.57	253.00	199.20	267.67				
Goods coverage	0.999	CK	0.997	0.997	0.998	0.997	0.997	<0.01	0.756	0.068	0.415
		L	0.997	0.997	0.998	0.997	0.997				
		Y	0.998	0.997	0.998	0.997	0.997				
		LY	0.998	0.996	0.997	0.998	0.997				
PD whole tree	16.64	CK	26.35	18.79	19.43	21.16	47.35	1.61	0.308	0.222	0.336
		L	20.16	22.73	18.15	18.82	28.97				
		Y	10.11	25.76	12.38	18.59	23.21				
		LY	15.03	22.42	28.69	17.48	24.00				

The CTPS was treated without (CK) or with *Lactobacillus buchneri* (L), yeast (Y), and a mixture of *L. buchneri* and yeast (LY); cellulase was added to each group; SEM, standard error of the mean; ^A-C^ indicate differences between values in the same column at *p* < 0.05; ^a-g^ indicate differences between values in the same row at *p* < 0.05.

### Bacterial community composition of CTPS during anaerobic storage

**Figure. 1:**
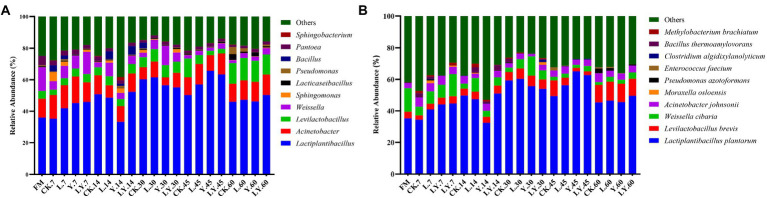
The relative abundance of dominant genera **(A)** and species **(B)** in cellulase-treated *Pennisetum sinese* (CTPS) after anaerobic storage. CK, control; L, *Lentilactobacillus buchneri*; Y, yeast; LY, a mixture of *L. buchneri* and yeast.

Changes in the bacterial community at the genus and species levels during the anaerobic storages are shown in Figure 1 and Table 5. At the genus level, the relative abundance of *Lactiplantibacillus* and *Levilactobacillus* increased with increasing storage time, but that of *Weissella* gradually decreased. Previous studies have shown that *Weissella* causes fermentation in the early stages, the predominant bacteria gradually shift to *Lactobacillus*, which is more tolerant to low pH (Cai et al., 1998). In the present study, the LY-treated CTPS had the highest relative abundance of *Weissella* (14.04%) after 7 days of anaerobic storage, which was also detected after 60 days of storage. The presence of *Weissella* at the late fermentation stage may be due to the high pH. In the present study, some *Bacillus* (relative richness >3.0%) was detected in the CK and LY treated CTPS in pre-fermentation. *Bacillus* accelerates lignocellulose degradation and promotes the stabilization and resource utilization of compost by secreting enzymes (Niu and Li, 2022). *Bacillus* probably correlated with the high lactic acid concentration of CK-and LY-treated CTPS in the pre-storage period. In addition, *Acinetobacter* also showed a high abundance in this study. It has been found that *Acinetobacter* can utilize acetic acid to survive in an anaerobic environment, and its abundance may increase with increasing acetic acid content (Ogunade et al., 2017). Thus, the acetic acid increase may be responsible for the higher abundance of *Acinetobacter*, the bacterium causes aerobic spoilage *via* the oxidation of lactic acid and acetic acid (Dolci et al., 2011). The high abundance of *Acinetobacter* during fermentation may also be responsible for the elevated pH after 30 days of fermentation.

**Table 5:** Relative abundance (%) of top four bacteria at different taxonomic levels in cellulase-treated *Pennisetum sinese* (CTPS) before and after anaerobic storage.

	Freshforage	Treatments (T)	Storage period (D)					SEM	*p*-value		
			Day 7	Day 14	Day 30	Day 45	Day 60		*T*	*D*	*T × D*
*Lactiplantibacillus*	35.88	CK	35.24	50.75	60.28	50.23	46.00	0.94	0.28	<0.001	0.054
		L	41.85	48.62	61.47	57.03	47.20				
		Y	45.11	33.23	56.48	65.72	46.15				
		LY	45.88	52.24	55.09	63.28	50.25				
*Acinetobacter*	12	CK	15.11ABa	12.05Ab	7.68ABc	11.38b	11.45b	0.28	0.566	<0.001	0.033
		L	14.72ABa	7.84Bb	10.02Aab	12.89a	12.44ab				
		Y	16.91Aa	10.06ABbc	4.95Bc	9.87bc	12.48ab				
		LY	12.48Bab	11.72Aab	9.30Ab	13.43a	13.00a				
*Levilactobacillus*	5.01	CK	3.25Bc	5.04bc	6.37b	11.84Aa	13.13a	0.23	0.187	<0.001	<0.001
		L	4.36ABb	5.77b	7.92b	5.76Bb	14.19a				
		Y	5.04Abc	4.44bc	7.81b	2.80Bc	13.43a				
		LY	5.28Ac	5.97bc	7.06b	2.91 Bd	12.77a				
*Weissella*	14.99	CK	5.53Ca	3.28Bb	2.76Bb	2.21ABb	1.80Bb	0.18	<0.001	<0.001	<0.001
		L	7.84Baa	8.58Aa	5.94Ba	2.60ABb	2.97ABb				
		Y	8.18Bb	3.86Bc	12.18Aa	1.91Bc	2.52ABc				
		LY	14.04Aa	5.20Bb	5.79Bb	3.41Ab	4.05Ab				
*Lactiplantibacillus plantarum*	35.18	CK	34.37Bc	49.69ab	59.36a	49.35Bab	45.34b	0.923	0.294	<0.001	0.0497
		L	40.88ABc	47.36bc	60.24a	56.26ABab	46.53bc				
		Y	44.07Aab	32.40b	55.59a	65.03Aa	45.59ab				
		LY	44.71Ac	50.96bc	53.96b	62.69Aa	49.52bc				
*Levilactobacillus brevis*	4.26	CK	2.73Bc	4.32b	5.34b	10.01Aa	11.07a	0.20	0.249	<0.001	<0.001
		L	3.66ABb	4.83b	6.68b	4.81Bb	12.01a				
		Y	4.28Abc	3.76bc	6.74b	2.26Bc	11.64a				
		LY	4.51Ac	5.08bc	5.92b	2.36 Bd	10.86a				
*Weissella cibaria*	14.99	CK	5.52Ca	3.28Bb	2.74Bb	2.21ABb	1.80Bb	0.18	<0.001	<0.001	<0.001
		L	7.84Ba	8.58Aa	5.93Ba	2.58ABb	2.96ABb				
		Y	8.17Bb	3.86c	12.17Aa	1.91Bc	2.51ABc				
		LY	14.03Aa	5.20Bb	5.80Bb	3.41Ab	4.05Ab				
*Acinetobacter johnsonii*	3.34	CK	5.89	4.81	2.83	3.96	5.55	0.13	0.16	<0.001	0.131
		L	5.17	2.88	3.39	4.51	3.98				
		Y	5.71	4.55	1.60	3.23	3.92				
		LY	4.65	4.14	3.59	3.99	4.37				

The CTPS was treated without (CK) or with *Lentilactobacillus buchneri* (L), yeast (Y), and a mixture of *L. buchneri* and yeast (LY); SEM, standard error of mean; ^A-C^ indicate differences between values in the same column at *p* < 0.05; ^a-g^ indicate differences between values in the same row at *p* < 0.05.

The top three abundant bacteria at the species level were *L. plantarum*, *L. brevis*, and *Weissella cibaria*. However, the study did not detect *L. buchneri* during storage, probably because the *L. buchneri* used in this study exerted low competition at the beginning of storage, which was replaced by *L. plantarum*. The high relative abundance of *L. plantarum* in storage supported this fact. Generally, *L. plantarum* promotes rapid fermentation that produces lactic acid, preventing further breakdown of the sugars and proteins (Yan et al., 2019). Thus, the lower relative abundance of *L. plantarum* in the CK-treated CTPS after 7 days of anaerobic storage explains the WSC content. Additionally, we found that *L. brevis* increased during storage in each treatment group. The Y treatment significantly increased the abundance of *L. brevis* at 7 days compared to the CK treatment, which may be due to the inoculated *S. cerevisiae* enhancing cellulose degradation by affecting the cellulase activity, thus providing more substrate for *L. brevis* growth. *L. brevis* is a heterologous fermenting bacterium that produces lactic acid and acetic acid as the primary end products through WSC metabolism (Tohno et al., 2012). The abundance of *L. brevis* also explains the increase in acetic acid content. Meanwhile, the relative abundance of *W. cibaria*, a heterologous fermenting bacterium, decreased with storage time in overall treated samples. *L. plantarum* significantly decreased the relative abundance of *Weissella*, which is usually outcompeted by *Lactobacillus* spp. as the pH declines during ensiling (Keshri et al., 2019). Interestingly, the LY-treated CTPS had the highest relative abundance of *W. cibaria* after storage for 60 days. This dominant *Weissella* resulted in the lowest alpha diversity in the LY-treated CTPS group at 60 days. Teixeira et al. (2021) reported that *W. cibaria* could ferment prebiotic fibers, which is related to β-glucosidase activity, suggesting that the *W. cibaria* during anaerobic storage could promote cellulose degradation. Therefore, we attribute the low cellulose content of the LY-treated CTPS in this study to the degradation of cellulose by *W. cibaria*. Unfortunately, the activity of cellulase was not determined in the current work. In summary, inoculation with *L. buchn*eir and *S. cerevisiae* increased the abundance of *L. brevis* and *W. cibaria,* which enhanced cellulose degradation.

### Functional prediction of bacterial community in CTPS during anaerobic storage

**Figure. 2:**
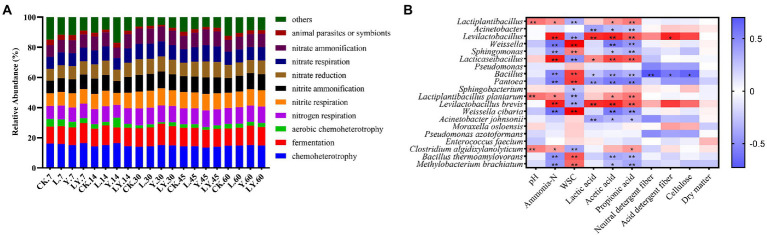
The functional prediction of bacteria **(A)** in cellulase-treated *Pennisetum sinese* (CTPS) during anaerobic storage and Spearman correlations between the fermentation parameters and the relative abundance of top 10 bacterial genera and species **(B)**. CK, control; L, *Lentilactobacillus buchneri*; Y, yeast; LY, a mixture of *L. buchneri* and yeast; WSC, water-soluble carbohydrates.

Figure 2A and Table 6 show the predicted functions of the bacterial community during anaerobic fermentation. Chemoheterotrophy and fermentation are ways in which microorganisms utilize organic matter, and their abundance represent the intensity of microbial activity. In the present, the functional group chemoheterotrophy was most abundant in the bacterial community, followed by fermentation and nitrogen-related cycling. The CTPS samples inoculated with LY, L, and Y demonstrated improved fermentation function at 7, 14, and 30 days, respectively. This improvement in function is attributed to the high relative abundance of *W. cibaria* (Table 5). Studies have proven that the presence of *W. cibaria* promotes anaerobic fermentation (Cai et al., 1998). In addition, higher nitrite ammonification, nitrogen respiration, and nitrite respiration were detected in the groups with or without the inoculants, which explains the persistent production of ammonia-N.

**Table 6:** Chemoheterotrophy and fermentation changes in functional prediction of bacteria in cellulase-treated *Pennisetum sinese* (CTPS) during anaerobic storage.

Items	Treatments (T)	Storage period (D)					SEM	*p*-value		
		Day 7	Day 14	Day 30	Day 45	Day 60		*T*	*D*	*T × D*
Chemoheterotrophy	CK	21.40	17.16	16.00	16.65	17.72	0.27	0.909	<0.001	0.088
	L	20.21	17.39	16.28	16.93	17.99				
	Y	18.84	22.95	16.22	15.31	17.52				
	LY	19.53	17.02	17.58	16.46	17.68				
Fermentation	CK	11.20B	11.71AB	12.25B	12.23	11.46	0.10	0.136	0.012	0.001
	L	11.88B	13.07A	12.56B	12.05	11.87				
	Y	11.75Bbc	10.34Bc	14.12Aa	11.81bc	12.72ab				
	LY	13.43Aa	12.15ABb	12.73Bab	11.8b	12.2b				
Nitrogen cycle	CK	48.65	58.26	64.18	58.67	55.79	0.66	0.539	0.012	0.143
	L	52.83	59.47	63.53	60.62	57.75				
	Y	54.02	46.52	63.47	64.67	57.91				
	LY	55.48	59.41	60.60	62.34	59.00				

The CTPS was treated without (CK) or with *Lentilactobacillus buchneri* (L), yeast (Y), and a mixture of *L. buchneri* and yeast (LY); SEM, standard error of the mean; ^A-C^ indicate differences between values in the same column at *p* < 0.05; ^a-g^ indicate differences between the values in the same row at *p* < 0.05.

**Figure. 3:**
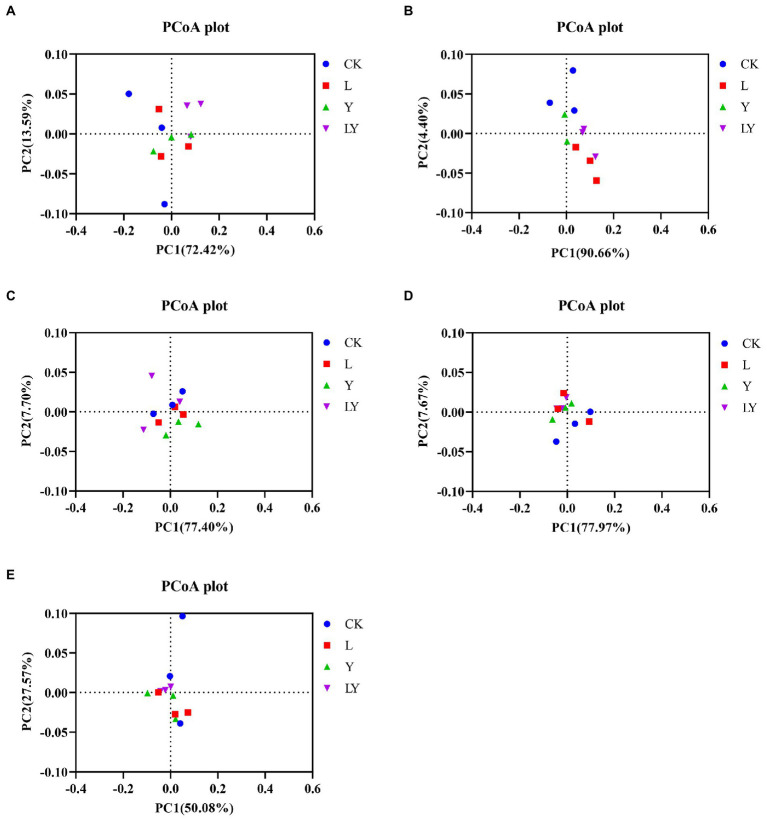
PCoA plot of the bacterial community structure of cellulase-treated *Pennisetum sinese* (CTPS) after anaerobic storage for 7 days **(A)**, 14 days **(B)**, 30 days **(C)**, 45 days **(D)**, and 60 days **(E)**. CK, control; L, *Lentilactobacillus buchneri*; Y, yeast; LY, a mixture of *L. buchneri* and yeast.

Finally, Spearman correlations between fermentation parameters and kinetics of the top ten genera and species during the fermentation of CTPS were calculated (Figure 2B). The analysis revealed that the pH was positively correlated with *Clostridium* and *L. plantarum*, suggesting that the variation in pH was not due to *L. plantarum* alone. Meanwhile, *Weissella* correlated negatively with ammonia-N but positively with WSC, implying that the presence of *Weissella* could preserve the nutritional quality of the anaerobic fermentation. Lactic acid and acetic acid showed positive correlations with *L. brevis*. Therefore, we inferred that *L. brevis* was the main component responsible for producing acetic acid during storage. Meanwhile, *Bacillus* showed a significant negative correlation with NDF, ADF, and cellulose, indicating the role of *Bacillus* in cellulose degradation, consistent with the reports on the ability of *Bacillus* to secrete cellulase (Niu and Li, 2022). Furthermore, to explore the effect of inoculants on the bacterial community we performed an analysis of bacterial PCoA. The PCoA plots illustrated that all inoculation promoted the change in the bacterial community during anaerobic fermentation (Figure 3). The LY-treated CTPS remodeled the bacterial community during storage significantly better than the other samples, indicating that *S. cerevisiae* resulted in a different effect on *L. buchneri*-mediated anaerobic fermentation.

## Conclusion

This study evaluated the effects of *L. buchneri*, *S. cerevisiae,* and their mixtures on cellulase treated *P. sinese* (CTPS) silage quality and PM microbial community. Among the several additives evaluated. *S. cerevisiae* appeared to be a potential inoculant, and its co-addition with LAB reduced the cellulose content of CTPS during anaerobic storage by increasing *W. cibaria* and *L. brevis*. This study showed that the combined addition of *S. cerevisiae* and *L. buchneri* reduced the cellulose content during anaerobic storage of CTPS.

## Data availability statement

The datasets presenting in this article are deposited in NCBI (https://www.ncbi.nlm.nih.gov/) repository, accession number PRJNA887958.

## Author contributions

CL: data curation, formal analysis, visualization, writing—original draft, writing—review and editing. XT, ML, GL, XH, LL, MZ: investigation. YX: investigation, resources. PL: conceptualization, methodology, validation, writing—review and editing, supervision, funding acquisition. CC: project administration, funding acquisition. All authors contributed to the article and approved the submitted version.

## Funding

This project was supported by the National Key Research and Development Program of China (2021YFD1300300) and the basic scientific research funds of Guizhou Province (2022).

## Conflict of interest

The authors declare that the research was conducted in the absence of any commercial or financial relationships that could be construed as a potential conflict of interest.

## Publisher’s note

All claims expressed in this article are solely those of the authors and do not necessarily represent those of their affiliated organizations, or those of the publisher, the editors and the reviewers. Any product that may be evaluated in this article, or claim that may be made by its manufacturer, is not guaranteed or endorsed by the publisher.

## References

[ref1] AOAC. (1990). Official Methods of Analysis. Association of Official Analytical Chemists, Arlington, VA.

[ref2] Broderick G. A. Kang J. H. (1980). Automated simultaneous determination of ammonia and total amino acids in ruminal fluid and in vitro media. J. Dairy Sci. 63, 64–75. doi: 10.3168/jds.S0022-0302(80)82888-8, PMID: 7372898

[ref3] Cai Y. Benno Y. Ogawa M. Ohmomo S. Kumai S. Nakase T. (1998). Influence of *lactobacillus* spp. from an inoculant and of *Weissella* and *Leuconostoc* spp. from forage crops on silage fermentation. Appl. Environ. Microbiol. 64, 2982–2987. doi: 10.1128/AEM.64.8.2982-2987.1998, PMID: 9687461 PMC106803

[ref4] Carvalho J. K. Panatta A. A. S. Silveira M. A. D. Tav C. Johann S. Rodrigues M. L. F. (2021). Yeasts isolated from a lotic continental environment in Brazil show potential to produce amylase, cellulase and protease. Biotechnol. Rep. 30:e00630. doi: 10.1016/j.btre.2021.e00630, PMID: 34136364 PMC8178091

[ref5] Chen L. Li P. Gou W. You M. Cheng Q. Bai S. (2020). Effects of inoculants on the fermentation characteristics and in vitro digestibility of reed canary grass (*Phalaris arundinacea* L.) silage on the Qinghai-Tibetan plateau. Anim. Sci. J. 91:e13364. doi: 10.1111/asj.13364, PMID: 32219952

[ref6] Colombatto D. Mould F. L. Bhat M. K. Phipps R. H. Owen E. (2004). In vitro evaluation of fibrolytic enzymes as additives for maize (*Zea mays* L.) silage. I. Effects of ensiling temperature, enzyme source and addition level. Anim. Feed Sci. Technol. 111, 111–128. doi: 10.1016/j.anifeedsci.2003.08.010

[ref7] Dolci P. Tabacco E. Cocolin L. Borreani G. (2011). Microbial dynamics during aerobic exposure of corn silage stored under oxygen barrier or polyethylene films. Appl. Environ. Microbiol. 77, 7499–7507. doi: 10.1128/AEM.05050-11, PMID: 21821764 PMC3209159

[ref8] Fujiwara M. Koyama M. Akizuki S. Ban S. Toda T. (2022). Influence of lignocellulosic components on the anaerobic digestibility of aquatic weeds: comparison with terrestrial crops. Ind. Crop. Prod. 178:114576. doi: 10.1016/j.indcrop.2022.114576

[ref9] Guo G. Yuan X. Li L. Wen A. Shao T. (2014). Effects of fibrolytic enzymes, molasses and lactic acid bacteria on fermentation quality of mixed silage of corn and hulless-barely straw in the Tibetan plateau. Grassl. Sci. 60, 240–246. doi: 10.1111/grs.12060

[ref10] Huang Z. Wang M. Ke W. Guo X. (2021). Screening of high 1,2-Propanediol production by *lactobacillus buchneri* strains and their effects on fermentation characteristics and aerobic stability of whole-plant corn silage. Agriculture 11:590. doi: 10.3390/agriculture11070590

[ref11] Keshri J. Chen Y. Pinto R. Kroupitski Y. Weinberg Z. G. Sela Saldinger S. (2019). Bacterial dynamics of wheat silage. Front. Microbiol. 10:1532. doi: 10.3389/fmicb.2019.01532, PMID: 31354651 PMC6632545

[ref12] Kung L. Shaver R. D. Grant R. J. Schmidt R. J. (2018). Silage review: interpretation of chemical, microbial, and organoleptic components of silages. J. Dairy Sci. 101, 4020–4033. doi: 10.3168/jds.2017-13909, PMID: 29685275

[ref13] Li D. Ni K. Zhang Y. Lin Y. Yang F. (2018b). Influence of lactic acid bacteria, cellulase, cellulase-producing *Bacillus pumilus* and their combinations on alfalfa silage quality. J. Integr. Agr. 17, 2768–2782. doi: 10.1016/S2095-3119(18)62060-X

[ref14] Li J. Yuan X. Dong Z. Mugabe W. Shao T. (2018a). The effects of fibrolytic enzymes, cellulolytic fungi and bacteria on the fermentation characteristics, structural carbohydrates degradation, and enzymatic conversion yields of *Pennisetum sinese* silage. Bioresour. Technol. 264, 123–130. doi: 10.1016/j.biortech.2018.05.059, PMID: 29800772

[ref15] Li P. Zhang Y. Gou W. Cheng Q. Bai S. Cai Y. (2019). Silage fermentation and bacterial community of bur clover, annual ryegrass and their mixtures prepared with microbial inoculant and chemical additive. Anim. Feed Sci. Tech. 247, 285–293. doi: 10.1016/j.anifeedsci.2018.11.009

[ref16] Li P. Zhao W. Yan L. Chen L. Chen Y. Gou W. (2022). Inclusion of abandoned rhubarb stalk enhanced anaerobic fermentation of alfalfa on the Qinghai Tibetan plateau. Bioresour. Technol. 347:126347. doi: 10.1016/j.biortech.2021.126347, PMID: 34808318

[ref17] Li M. Zhou H. L. Zi X. J. Cai Y. M. (2017). Silage fermentation and ruminal degradation of stylo prepared with lactic acid bacteria and cellulase. Anim. Sci. J. 88, 1531–1537. doi: 10.1111/asj.12795, PMID: 28402051

[ref18] Lin M. Feng L. Cheng Z. Wang K. (2021). Effect of ethanol or lactic acid on volatile fatty acid profile and microbial community in short-term sequentially transfers by ruminal fermented with wheat straw in vitro. Process Biochem. 102, 369–375. doi: 10.1016/j.procbio.2020.12.018

[ref19] Matano Y. Hasunuma T. Kondo A. (2013). Simultaneous improvement of saccharification and ethanol production from crystalline cellulose by alleviation of irreversible adsorption of cellulase with a cell surface-engineered yeast strain. Appl. Microbiol. Biot. 97, 2231–2237. doi: 10.1007/s00253-012-4587-x, PMID: 23184221

[ref20] McDonald P. Henderson N. Heron S. (1991). The biochemistry of silage. Chalcombe Publications, Marlow.

[ref21] Moharrery A. Hvelplund T. Weisbjerg M. R. (2009). Effect of forage type, harvesting time and exogenous enzyme application on degradation characteristics measured using in vitro technique. Anim. Feed Sci. Tech. 153, 178–192. doi: 10.1016/j.anifeedsci.2009.06.001

[ref22] Mu L. Xie Z. Hu L. Chen G. Zhang Z. (2020). Cellulase interacts with *lactobacillus plantarum* to affect chemical composition, bacterial communities, and aerobic stability in mixed silage of high-moisture amaranth and rice straw. Bioresour. Technol. 315:123772. doi: 10.1016/j.biortech.2020.123772, PMID: 32653750

[ref23] Niu J. Li X. (2022). Effects of microbial inoculation with different indigenous *bacillus* species on physicochemical characteristics and bacterial succession during short-term composting. Fermentation 8:152. doi: 10.3390/fermentation8040152

[ref24] Ogunade I. M. Jiang Y. Kim D. H. Cervantes A. A. P. Arriola K. G. Vyas D. (2017). Fate of *Escherichia coli* O157:H7 and bacterial diversity in corn silage contaminated with the pathogen and treated with chemical or microbial additives. J. Dairy Sci. 100, 1780–1794. doi: 10.3168/jds.2016-11745 28041727

[ref25] Patinvoh R. J. Osadolor O. A. Chandolias K. Sárvári Horváth I. Taherzadeh M. J. (2017). Innovative pretreatment strategies for biogas production. Bioresour. Technol. 224, 13–24. doi: 10.1016/j.biortech.2016.11.083, PMID: 27908585

[ref26] Ren F. He R. Zhou X. Gu Q. Xia Z. Liang M. (2019). Dynamic changes in fermentation profiles and bacterial community composition during sugarcane top silage fermentation: a preliminary study. Bioresour. Technol. 285:121315. doi: 10.1016/j.biortech.2019.121315, PMID: 30965280

[ref27] Steinbrenner J. Nägele H. Buschmann A. Hülsemann B. Oechsner H. (2019). Testing different ensiling parameters to increase butyric acid concentration for maize silage, followed by silage separation and methane yield potential of separated solids residues. Bioresour. Technol. Rep. 7:100193. doi: 10.1016/j.biteb.2019.100193

[ref28] Stokes M. R. (1992). Effects of an enzyme mixture, an inoculant, and their interaction on silage fermentation and dairy production. J. Dairy Sci. 75, 764–773. doi: 10.3168/jds.S0022-0302(92)77814-x, PMID: 1569268

[ref29] Teixeira C. G. Fusieger A. Milião G. L. Martins E. Drider D. Nero L. A. (2021). *Weissella*: an emerging bacterium with promising health benefits. Probiotics Antimicrob. Proteins 13, 915–925. doi: 10.1007/s12602-021-09751-1, PMID: 33565028

[ref30] Tohno M. Kobayashi H. Nomura M. Kitahara M. Ohkuma M. Uegaki R. (2012). Genotypic and phenotypic characterization of lactic acid bacteria isolated from Italian ryegrass silage. Anim. Sci. J. 83, 111–120. doi: 10.1111/j.1740-0929.2011.00923.x, PMID: 22339691

[ref31] Van Soest P. J. Robertson J. B. Lewis B. A. (1991). Methods for dietary fiber, neutral detergent fiber, and non-starch-polysaccharides in relation to animal nutrition. J. Dairy Sci. 74, 3583–3597. doi: 10.3168/jds.S0022-0302(91)78551-2, PMID: 1660498

[ref32] Wang C. Zhang J. Hu F. Zhang S. Lu J. Liu S. (2020). Bio-pretreatment promote hydrolysis and acidification of oilseed rape straw: roles of fermentation broth and micro-oxygen. Bioresour. Technol. 308:123272. doi: 10.1016/j.biortech.2020.123272, PMID: 32276202

[ref33] Wayne Polley H. Wilsey B. J. Derner J. D. (2007). Dominant species constrain effects of species diversity on temporal variability in biomass production of tallgrass prairie. Oikos 116, 2044–2052. doi: 10.1111/j.2007.0030-1299.16080.x

[ref34] Wu D. Li L. Zhao X. Peng Y. Yang P. Peng X. (2019). Anaerobic digestion: a review on process monitoring. Renew. Sust. Energ. Rev. 103, 1–12. doi: 10.1016/j.rser.2018.12.039

[ref35] Xu Z. He H. Zhang S. Guo T. Kong J. (2017). Characterization of feruloyl esterases produced by the four *lactobacillus* species: *L. amylovorus*, *L. acidophilus*, *L. farciminis* and *L. fermentum*, isolated from ensiled corn Stover. Front. Microbiol. 8, 1–11. doi: 10.3389/fmicb.2017.00941, PMID: 28626449 PMC5454770

[ref36] Yan Y. Li X. Guan H. Huang L. Ma X. Peng Y. (2019). Microbial community and fermentation characteristic of Italian ryegrass silage prepared with corn Stover and lactic acid bacteria. Bioresour. Technol. 279, 166–173. doi: 10.1016/j.biortech.2019.01.107, PMID: 30721817

[ref37] Zhang J. Kawamoto H. Cai Y. (2010). Relationships between the addition rates of cellulase or glucose and silage fermentation at different temperatures. Anim. Sci. J. 81, 325–330. doi: 10.1111/j.1740-0929.2010.00745.x, PMID: 20597889

[ref38] Zhou Y. Drouin P. Lafrenière C. (2016). Effect of temperature (5-25°C) on epiphytic lactic acid bacteria populations and fermentation of whole-plant corn silage. J. Appl. Microbiol. 121, 657–671. doi: 10.1111/jam.13198, PMID: 27271320

[ref39] Zielinska K. Fabiszewska A. Stefanska I. (2015). Different aspects of *lactobacillus* inoculants on the improvement of quality and safety of alfalfa silage. Chil. J. Agr. Res. 75, 298–306. doi: 10.4067/S0718-58392015000400005

